# Supplementary figures and images for: Patterns and predictors of sick leave among Swedish non-hospitalized healthcare and residential care workers with Covid-19 during the early phase of the pandemic
Source: PLoS One. 2021 Dec 9;16(12):e0260652. doi: 10.1371/journal.pone.0260652 (PMC8659339; doi:10.1371/journal.pone.0260652)

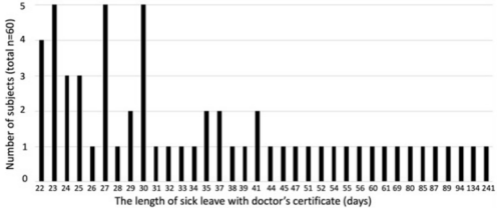

Supplement: S1 Fig — (PDF) [file pone.0260652.s001.pdf]
