# Supplementary material for: Patterns and predictors of sick leave among Swedish non-hospitalized healthcare and residential care workers with Covid-19 during the early phase of the pandemic
Source: PLoS One. 2021 Dec 9;16(12):e0260652. doi: 10.1371/journal.pone.0260652 (PMC8659339; doi:10.1371/journal.pone.0260652)
Supplement: Attachment — Submitted filename: PONE-D-21-21612_mrg_review.docx [file pone.0260652.s002.docx]

**PLOS ONE**

**Patterns and predictors of sick leave among non-hospitalized healthcare and residential care workers with Covid-19 during the early phase of the pandemic**

--Manuscript Draft--

| **Manuscript Number:** | PONE-D-21-21612 |
| --- | --- |
| **Article Type:** | Research Article |
| **Full Title:** | Patterns and predictors of sick leave among non-hospitalized healthcare and residential care workers with Covid-19 during the early phase of the pandemic |
| **Short Title:** | Sick leave due to Covid-19 among non-hospitalized healthcare and residential care |
| **Corresponding Author:** | Marta A Kisiel, M.D., Ph.D.  Uppsala University/University Hospital Aakademiska Uppsala, SWEDEN |
| **Keywords:** | predictors of sick leave due to covid-19, healthcare and residential care |
| **Abstract:** | Background  Healthcare and residential care workers represent two occupational groups that have, in particular, been at risk of Covid-19, its long-term consequences, and related sick leave. In this study, we investigated the predictors of sick leave among healthcare and residential workers due to non-hospitalized Covid-19 in the early period of the pandemic.  Materials and methods  This study is based on a patient register (n=3209) and included non-hospitalized healthcare or residential care service workers with a positive RT- PCR for SARS-CoV- 2 (n=433) between March and August 2020. Data such as socio-demographics, clinical characteristics, and the length of sick leave because of Covid-19 and prior to the pandemic were extracted from the patient’s electronic health records. Based on the Swedish pandemic policy, the study population was divided into two groups with sick leave ≤ 3 weeks and > 3 weeks, and longer sick leave was predicted by a generalized linear model with a binary distribution adjusted for age, gender, and comorbidity.  Results  Of 433 (77% women) healthcare and residential care workers included in this study, 14.8% needed longer sick leave (> 3 weeks) due to Covid-19. Only 1.4% of the subjects were on sick leave because of long Covid. The risk of sick leave was increased two-fold among residential care workers (adjusted RR 2.14 [95% CI 1.31–3.51]). Depression/anxiety (adjusted RR 2.09 [95% CI 1.31–3.34]), obesity (adjusted RR 1.96 [95% CI 1.01–3.81]) and dyspnea at symptom onset (adjusted RR  2.47 [95% CI 1.55–3.92]), sick leave prior to the pandemic (3–12 weeks) (adjusted RR  2.23 [95% CI 1.21–4.10) were associated with longer sick leave. Conclusion  In a public health perspective considering occupational category, comorbidity, symptom at onset, and sick leave prior to the pandemic might be important predictors of sick leave in healthcare, which can help prevent staff shortage. |
| **Order of Authors:** | Marta A Kisiel, M.D., Ph.D. |
|  | Tobias Nordquist |
|  | Gabriel Westman |
|  | Magnus Svartegren |
|  | Andrei Malinovschi |
|  | Helena Janols |
| **Additional Information:** |  |
| **Question** | **Response** |
| **Financial Disclosure** | MAK received funding for this work, a resident research program (held by Marta A. Kisiel) at the Uppsala University Hospital. |

| Enter a financial disclosure statement that describes the sources of funding for the work included in this submission. Review the [submission guidelines](http://journals.plos.org/plosone/s/submission-guidelines#loc-financial-disclosure-statement) for detailed requirements. View published research articles from [*PLOS ONE*](http://journals.plos.org/plosone/) for specific examples.  This statement is required for submission and **will appear in the published article** if the submission is accepted. Please make sure it is accurate. |  |
| --- | --- |
| **Unfunded studies**  Enter: *The author(s) received no specific funding for this work.*  **Funded studies**  Enter a statement with the following details:   - Initials of the authors who received each award - Grant numbers awarded to each author - The full name of each funder - URL of each funder website - Did the sponsors or funders play any role in the study design, data collection and analysis, decision to publish, or preparation of the manuscript? - **NO** - Include this sentence at the end of your statement: *The funders had no role in study design, data collection and analysis, decision to publish, or preparation of the manuscript.* - **YES** - Specify the role(s) played. |  |
| * typeset |  |
| **Competing Interests**  Use the instructions below to enter a competing interest statement for this submission. On behalf of all authors, disclose any [competing interests](http://journals.plos.org/plosone/s/competing-interests) that could be perceived to bias this work—acknowledging all financial support and any other relevant financial or non- financial competing interests.  This statement is required for submission and **will appear in the published article** if the submission is accepted. Please make | The authors have declared that no competing interests exist. |

| sure it is accurate and that any funding sources listed in your Funding Information later in the submission form are also declared in your Financial Disclosure statement.  View published research articles from  [*PLOS ONE*](http://journals.plos.org/plosone/) for specific examples. |  |
| --- | --- |
| **NO authors have competing interests**  Enter: *The authors have declared that no competing interests exist.*  **Authors with competing interests**  Enter competing interest details beginning with this statement:  *I have read the journal's policy and the authors of this manuscript have the following competing interests: [insert competing interests here]* |  |
| * typeset |  |
| **Ethics Statement**  Enter an ethics statement for this submission. This statement is required if the study involved:   - Human participants - Human specimens or tissue - Vertebrate animals or cephalopods - Vertebrate embryos or tissues - Field research   Write "N/A" if the submission does not require an ethics statement.  General guidance is provided below. Consult the [submission guidelines](http://journals.plos.org/plosone/s/submission-guidelines#loc-guidelines-for-specific-study-types) for detailed instructions. **Make sure that all information entered here is included in the Methods section of the manuscript.** | The study was approved by the institutional ethics committee of the University Hospital in Uppsala (2020-05707) and conducted in accordance with the Helsinki Declaration. All medi-cal records were anonymized; only statistical information was used for research purposes. |

| **Format for specific study types**  **Human Subject Research (involving human participants and/or tissue)**   - Give the name of the institutional review board or ethics committee that approved the study - Include the approval number and/or a statement indicating approval of this research - Indicate the form of consent obtained (written/oral) or the reason that consent was not obtained (e.g. the data were analyzed anonymously)   **Animal Research (involving vertebrate animals, embryos or tissues)**   - Provide the name of the Institutional Animal   Care and Use Committee (IACUC) or other relevant ethics board that reviewed the study protocol, and indicate whether they approved this research or granted a formal waiver of ethical approval   - Include an approval number if one was obtained - If the study involved *non-human primates*, add *additional details* about animal welfare and steps taken to ameliorate suffering - If anesthesia, euthanasia, or any kind of animal sacrifice is part of the study, include briefly which substances and/or methods were applied   **Field Research**  Include the following details if this study involves the collection of plant, animal, or other materials from a natural setting:   - Field permit number - Name of the institution or relevant body that granted permission |  |
| --- | --- |
|  |  |
| **Data Availability**  Authors are required to make all data underlying the findings described fully available, without restriction, and from the time of publication. PLOS allows rare exceptions to address legal and ethical concerns. See the [PLOS Data Policy](http://journals.plos.org/plosone/s/data-availability) and [FAQ](http://journals.plos.org/plosone/s/data-availability#loc-faqs-for-data-policy) for detailed information. | Yes - all data are fully available without restriction |

| A Data Availability Statement describing where the data can be found is required at submission. Your answers to this question constitute the Data Availability Statement and **will be published in the article**, if accepted. |  |
| --- | --- |
| **Important:** Stating ‘data available on request from the author’ is not sufficient. If your data are only available upon request, select ‘No’ for the first question and explain your exceptional situation in the text box. |  |
| Do the authors confirm that all data underlying the findings described in their manuscript are fully available without restriction? |  |
| **Describe where the data may be found in full sentences. If you are copying our sample text, replace any instances of XXX with the appropriate details.**   - If the data are **held or will be held in a public repository**, include URLs, accession numbers or DOIs. If this information will only be available after acceptance, indicate this by ticking the box below. For example: *All XXX files are available from the XXX database (accession number(s) XXX, XXX.*). - If the data are all contained **within the manuscript and/or Supporting Information files**, enter the following: *All relevant data are within the manuscript and its Supporting Information files.* - If neither of these applies but you are able to provide **details of access elsewhere**, with or without limitations, please do so. For example:   *Data cannot be shared publicly because of [XXX]. Data are available from the XXX Institutional Data Access / Ethics Committee (contact via XXX) for researchers who meet the criteria for access to confidential data.*  *The data underlying the results presented in the study are available from (include the name of the third party* | All data is available and handled by the authors. All data can not be shared publicly as it contain personalnumber but it can be unidentified and presented as a group analysis. |

| *and contact information or URL).*   - This text is appropriate if the data are owned by a third party and authors do not have permission to share the data.   * typeset |  |
| --- | --- |
| Additional data availability information: | Tick here if the URLs/accession numbers/DOIs will be available only after acceptance of the manuscript for publication so that we can ensure their inclusion before publication. |

Manus	[Click here to access/download;Manuscript;210623_MKisiel Predictors of sick leave (korr).doc](https://www.editorialmanager.com/pone/download.aspx?id=29369669&amp;guid=7b28fd5d-14bb-4a3b-ac35-d0e1b5a1d567&amp;scheme=1)

# Patterns and predictors of sick leave among non-hospitalized healthcare and residential care workers with Covid-19 during the early phase of the pandemic

Marta A. Kisiel1* M.D. Ph.D., Tobias Nordqvist1 Ph.D., Gabriel Westman2 M.D., Ph.D., Mag- nus Svartegren1 M.D., Ph.D., Andrei Malinovschi3 M.D., Ph.D., Helena Janols2 M.D. Ph.D.

1. Department of Medical Sciences, Occupational and Environment Medicine, Uppsala University, Uppsala, Sweden
2. Department of Medical Sciences, Infectious Diseases, Uppsala University, Uppsala, Sweden
3. Department of Medical Sciences, Clinical Physiology, Uppsala University, Uppsala, Sweden

*Corresponding author, Dag Hammarskjölds väg 60, 751 85 Uppsala

# Abstract

Background

Healthcare and residential care workers represent two occupational groups that have, in par- ticular, been at risk of Covid-19, its long-term consequences, and related sick leave. In this study, we investigated the predictors of prolonged sick leave among healthcare and residential workers due to non-hospitalized Covid-19 in the early period of the pandemic.

Materials and methods

This study is based on a patient register (n=3209) and included non-hospitalized healthcare or residential care service workers with a positive RT- PCR for SARS-CoV-2 (n=433) between March and August 2020. Data such as socio-demographics, clinical characteristics, and the length of sick leave because of Covid-19 and prior to the pandemic were extracted from the patient’s electronic health records. Based on the Swedish pandemic policy, the study popula- tion was divided into two groups with sick leave ≤ 3 weeks and > 3 weeks, and longer sick leave was predicted by a generalized linear model with a binary distribution adjusted for age, gender, and comorbidity.

Results

Of 433 (77% women) healthcare and residential care workers included in this study, 14.8% needed longer sick leave (> 3 weeks) due to Covid-19. Only 1.4% of the subjects were on sick leave because of long Covid. The risk of sick leave was increased two-fold among residential care workers (adjusted RR 2.14 [95% CI 1.31–3.51]). Depression/anxiety (adjusted RR 2.09

[95% CI 1.31–3.34]), obesity (adjusted RR 1.96 [95% CI 1.01–3.81]) and dyspnea at symp- tom onset (adjusted RR 2.47 [95% CI 1.55–3.92]), sick leave prior to the pandemic (3–12 weeks) (adjusted RR 2.23 [95% CI 1.21–4.10]) were associated with longer sick leave.

Conclusion

From a public health perspective, considering occupational category, comorbidity, symptoms at onset, and sick leave prior to the pandemic as potential predictors of sick leave in healthcare may help prevent staff shortage.

# Introduction

Healthcare and residential care workers had the highest rate of SARS-CoV-2 positive cases compared to other occupational groups, particularly in the first wave of the pandemic [1-4]. A large population study from the UK found a seven-fold increased risk of Covid-19 among healthcare and two-fold higher risk for residential care workers [5]. Risk factors for Covid-19 among healthcare and residential care workers included inadequate personal protective equipment, suboptimal handwashing, and working long hours in high-risk departments [6-9].

Even though the majority of healthcare personnel had mild disease [1, 10], up to 10% might have had symptoms that persisted beyond 12 weeks, called long Covid, reducing their work ability [11]. Studies from the US and Spain showed that healthcare workers also had an increased prevalence of sick leave during the pandemic [12, 13], a major driver of high healthcare costs [14]. Sick leave has a multifactorial association between morbidity, education, working conditions, job satisfaction, general health and family life—as an indicator of individuals’ well-being, but also a predictor of health consequences [15-17].

In Sweden, it was estimated that 3.4% of healthcare and 1.3% of residential care workers (per 100,000 people) had Covid-19 in the first wave of the pandemic [18]. Factors such as hospitalization, higher age, and sick leave prior to the pandemic were the risk factors of longer sick leave in the Swedish general population [5].

A recent editorial by Gohar suggesting that staff shortage, increased work demand, personal factors such as age, work experience, job role, health, history of previous sick leave, the or- ganization safety and job support employees were the predictive factors for sick leave in

healthcare [19]. In this study, we investigated predictors of prolonged sick leave after having a confirmed Covid-19 illness among non-hospitalized healthcare and residential care workers. The study covers the early period of the pandemic, where testing of both occupational categories was prioritized, providing valuable clinical and epidemiological information.

# Materials and methods

Study design

This study, being a part of COMBAT post Covid project investigating long-term consequences of non-hospitalized Covid-19, is based on a patient register (n=3209) contained symptomatic individuals who were tested at one Covid-19 testing outpatient created at

the Department of Infectious Disease of the Uppsala University Hospital between March 10th and August 21st, 2020. This study population included symptomatic employees of Region Uppsala who tested positive for SARS-CoV-2 by reverse transcription–polymerase chain re- action (RT-PCR) on nasopharyngeal swab, not receiving in-patient care, and working

in healthcare or residential care service. The included subjects were followed up after 8-12 months (until the end of April 2021). The Region Uppsala includes Uppsala University Hos- pital, primary healthcare including residential care service, and primary care centers. Positive RT-PCR was the basis for a confirmed diagnosis of Covid-19 (U07.1), in accordance with the International Statistical Classification of Disease (ICD) (Please see the link: https://icd.who.int/browse10/2019/en#/U07.1).

The information for this study was further collected from the electronic medical records that contain all information on patients readily available for healthcare providers in the Uppsala Region. The study population was divided into two groups (≤ 3 weeks and > 3 weeks) based on the length of sick leave due to confirmed Covid-19, in line with the Swedish pandemic policy on sick leave. In line with this policy, all suspected and confirmed Covid-19 cases were advised to self-isolate, having a paid sick leave up to 21 days (≤ 3 weeks) from the symptom onset. From day 22 (> 3 weeks), a doctor’s certificate was required for prolonged paid sick leave [20]. The group with sick leave > 3 weeks, defined as longer sick leave, included subjects who required a doctor’s certificate. Some subjects have more than one doc- tor’s certificate for sick leave because of Covid-19, but there were no time gaps between them. Therefore, the length of sick leave in the group > 3 weeks was calculated as a period between a day of positive RT-PCR result and the last day of the doctor’s certificate. We also assessed sick leave > 12 weeks, called long Covid, in accordance with the WHO’s definition of long Covid, which is long-term symptoms after the infection beyond 12 weeks (Please see also: https:/[/www.who.int/publi](http://www.who.int/publications/i/item/WHO-2019-nCoV-clinical-2021-1))c[ations/i/item/WHO-2019-nCoV-clinical-2021-1).](http://www.who.int/publications/i/item/WHO-2019-nCoV-clinical-2021-1))

The study was approved by the institutional ethics committee of the University Hospital in Uppsala (2020-05707) and conducted in accordance with the Helsinki Declaration. All medi- cal records were anonymized; only statistical information was used for research purposes.

Variables

Besides information on sick leave as a result of Covid-19, we collected the following data from the patient’s medical records:

- Age (in years) and sex (woman/man).
- Occupation, categorized as physician, nurse/midwives, assistant nurse, psychologist/counselor, biomedical analytics, occupational- and physiotherapist, residential care workers, health supporting staff such as medical administrators, laboratory workers, maintenance workers.
- Symptom at onset such as fever, dyspnea, muscle and joint pain, insomnia/ageusia, sore throat, headache, congestion, fatigue, pressure over chest/chest pain.
- History of common chronic disease including diabetes, hypertension, other heart disease, chronic lung disease including asthma and obstructive lung disease, previous or current cancer/immunosuppressive treatment, hypo/hyperthyreosis, previous or current depression/anxiety.
- BMI in kg/m^2^, classified as underweight < 18.5, normal 18.50 to 25.00, overweight

25.01 to 30.00, and obesity > 30.

- Sick leave prior to the pandemic, defined as sick leave with a doctor’s certificate from January 1, 2019 and February 28, 2020. In case of more than one sick leave period, the length was summed together and categorized as ≤ 3 weeks, 3–12 weeks, > 12 weeks of sick leave.

Statistical analysis

Data were summarized using frequency, percentage, mean, and standard deviation. Differences between the groups (≤ 3 weeks and > 3 weeks sick leave) were assessed using Chi2 test or Fisher’s exact test (if the assumption for Chi2 test was not met). P < 0.05 was considered statistically significant.

Risk ratios (RR) and 95% confidence intervals (95% CI) adjusted for age, gender and comorbidity were calculated using a multivariable generalized linear model to examine the factors predicting longer sick leave (> 3 weeks). Covariates included in the model were chosen based on clinical and theoretical reasoning. All analysis was managed in Excel and SAS 9.4.

# Results

Socio-demographic and clinical characteristics

This study included 433 non-hospitalized subjects with detectable SARS-CoV-2 by RT-PCR, representing healthcare and residential care workers, of which 335 (77%) were women. Sixty individuals (13.8%) had longer sick leave (> 3 weeks) due to confirmed Covid-19. All basic and clinical characteristics of the study population are shown in Table 1. The length of the complementary doctor’s certificate was between 6–134 days (mean 43 days), Supplementary figure 1. One subject was on sick leave at the end of the end of the study period (241 days). Six subjects (1.4%) were on sick leave for > 12 weeks.

Predictors of prolonged sick leave

Age and sex had no association with longer sick leave. Residential care workers had twice the risk of long sick leave because of Covid-19 (adjusted RR 2.14 [95% CI 1.31–3.51], while the risk of sick leave was not increased in other healthcare categories. Among comorbidities, obesity (adjusted RR 1.96 [95% CI 1.01–3.81]) and depression/anxiety (adjusted RR 2.09 [95% CI 1.31–3.34]) were significantly associated with longer sick leave due to Covid-19. We did not consider the relative risk of being underweight since the number of samples in the group > 3 weeks was low. The presence of dyspnea at symptom onset (adjusted RR 2.47 [95% CI 1.55–3.92]) was the only symptom that predicted longer sick leave. Sick leave prior to the

pandemic (3–12 weeks) led to an increased risk of sick absence as a result of Covid-19, Figure 1.

# Discussion

To the best of our knowledge, this is the first study investigating the pattern and predictors of sick leave among healthcare and residential care workers with non-hospitalized confirmed Covid-19 in the early period of the pandemic. Our main result was that working at residential care exhibited almost two-fold higher risk for longer sick leave (< 3 weeks), while other healthcare groups had no elevated risk of sick absence due to Covid-19. Our finding is in line with research conducted prior to the pandemic showing that residential care workers had the highest number of cases with long-term sickness absence compared to other professional categories [21]. In those studies, identified risk factors included: having a stressful work environment, poor work support, shortage of staff, increased mental and physical fatigue, and low job control [19, 22, 23]. In Sweden, the media highlighted that at the beginning of the pandemic, the work demand was increased, and there was limited availability of protective equipment in residential care service [24, 25].

We found that depression and anxiety were predictors of prolonged sick leave. A study from China showed that approximately one-fifth of hospitalized Covid-19 patients had depression and anxiety [26]. A study conducted prior to the pandemic found that mental health disease was an important factor in longer and repeated sick leave, particularly in younger men and women [27, 28].

As illustrated in our generalized linear model, obesity (> 30) was linked to a greater risk of longer sick absence. This finding is congruent with several previous studies showing that obese individuals have a higher likelihood of sickness absence compared to normal weight individuals [29, 30]. Obesity was also associated with increased risk of hospitalization due to Covid-19, and critical outcomes of the infection, particularly in younger people [31-33].

Having dyspnea as a baseline symptom was a factor that predicted longer sick leave in our study. This is in line with a previous study where dyspnea at baseline was the only predictor affecting the recovery, both in outpatients and admitted patients [34]. The meta-analysis on Covid-19 showed that dyspnea as a symptom onset was strongly associated with progression of Covid-19 and risk of hospitalization [35].

Further, our study found that 3–12 weeks of sick leave prior to the pandemic increased the risk of prolonged sick absence due to Covid-19 infection. This effect was not found in those with > 12 weeks of sick leave prior to the pandemic, which might be because of the low sample of subjects. A recent study on sick leave as a result of Covid-19 in the general population reported that sick leave prior to the pandemic was linked to a longer sick absence due to Covid-19 [36]. Also, studies conducted prior to the pandemic showed that healthcare workers who had been on previous sick leave were also at risk of future absenteeism [23, 37, 38].

In this study, we found that sick leave because of long Covid (> 12 weeks) was uncommon and affected less than 2% of the studied non-hospitalized healthcare and residential care workers. In contrast, a study on the Swedish general population, based on register data, showed that long Covid affected 13% of individuals in the early time of the pandemic [36]. This study included both hospitalized and non-hospitalized individuals of different occupations. Also, the previous study on Swedish healthcare showed that eight months after mild

Covid-19, one in ten individuals still had at least one moderate to severe symptoms, long Covid, which negatively impacted their daily life and work ability [11].

The study has several strengths. It is based on all healthcare and residential care workers with confirmed Covid-19, tested during the first wave of the pandemic in the same testing center in Uppsala. Clinical characteristics were collected from the medical records, which reduces bias in comparison to self-reported information. By using patients’ medical records, instead of the Swedish Insurance System, sick leave information was assessed in real time (until the end of April 2021) without delay. However, a limitation is that there might be a bias by collecting sick leave information in this way as Swedish inhabitants are free to seek health care in different regions that have distinct patient records. BMI was missing in the substantial part of the study population. We had no information on education, which may be important to consider while studying sick leave. However, we found no effects of sick leave among assistant nurses from primary and secondary care that had a similar educational level to residential care workers.

Conclusions

In this study we showed that working in residential care, obesity, depression/anxiety, and longer sick leave prior to the pandemic were predictors of sick leave in confirmed Covid- 19 non hospitalized healthcare cases. The pandemic is not over yet and, from a public health perspective, it might be important to consider strategies to both avoid staff shortage and develop proper rehabilitation for affected individuals among front-line workers.

# Acknowledgments

The authors would like to acknowledge medical students Tove Wikström and Hanna Broman for helping with the data collection.

# Financial Disclosure Statement and competing interests

This study was founded by a resident research program (held by Marta A. Kisiel) at the Upp- sala University Hospital. All authors report no conflict of interests.

References

1. Gomez-Ochoa, S.A., et al., *COVID-19 in Health-Care Workers: A Living Systematic Review and Meta-Analysis of Prevalence, Risk Factors, Clinical Characteristics, and Outcomes.* Am J Epidemiol, 2021. **190**(1): p. 161-175.
2. Pouwels, K.B., et al., *Community prevalence of SARS-CoV-2 in England from April to November, 2020: results from the ONS Coronavirus Infection Survey.* Lancet Public Health, 2021. **6**(1): p. e30-e38.
3. Lan, F.Y., et al., *Work-related COVID-19 transmission in six Asian countries/areas: A follow-up study.* PLoS One, 2020. **15**(5): p. e0233588.
4. Jin, H., et al., *Novel coronavirus pneumonia emergency in Zhuhai: impact and challenges.* J Hosp Infect, 2020. **104**(4): p. 452-453.
5. Mutambudzi, M., et al., *Occupation and risk of severe COVID-19: prospective cohort study of 120 075 UK Biobank participants.* Occup Environ Med, 2020.
6. Li, W., et al., *An evaluation of COVID-19 transmission control in Wenzhou using a modified SEIR model.* Epidemiol Infect, 2021. **149**: p. e2.
7. Adelina Comas-Herrera (CPEC, L.a.J.Z.S., *Mortality associated with COVID-19 outbreaks in care homes: early international evidence*. 2020: International long term care policy netweork
8. Ran, L., et al., *Risk Factors of Healthcare Workers With Coronavirus Disease 2019: A Retrospective Cohort Study in a Designated Hospital of Wuhan in China.* Clin Infect Dis, 2020. **71**(16): p. 2218-2221.
9. Wang, X., et al., *Risk factors of SARS-CoV-2 infection in healthcare workers: a retrospective study of a nosocomial outbreak.* Sleep Med X, 2020. **2**: p. 100028.
10. Sahu, A.K., et al., *COVID-19 in health care workers - A systematic review and meta- analysis.* Am J Emerg Med, 2020. **38**(9): p. 1727-1731.
11. Havervall, S., et al., *Symptoms and Functional Impairment Assessed 8 Months After Mild COVID-19 Among Health Care Workers.* JAMA, 2021. **325**(19): p. 2015-2016.
12. Groenewold, M.R., et al., *Increases in Health-Related Workplace Absenteeism Among Workers in Essential Critical Infrastructure Occupations During the COVID-19 Pandemic - United States, March-April 2020.* MMWR Morb Mortal Wkly Rep, 2020. **69**(27): p. 853-858.
13. Calvo-Bonacho, E., et al., *COVID-19 and Sick Leave: An Analysis of the Ibermutua Cohort of Over 1,651,305 Spanish Workers in the First Trimester of 2020.* Front Public Health, 2020. **8**: p. 580546.
14. Maltezou, H.C., et al., *Costs associated with COVID-19 in healthcare personnel in Greece: a cost-of-illness analysis.* J Hosp Infect, 2021.
15. Amiri, S. and S. Behnezhad, *Association between job strain and sick leave: a systematic review and meta-analysis of prospective cohort studies.* Public Health, 2020. **185**: p. 235-242.
16. Donders, N.C., et al., *Age differences in the associations between sick leave and aspects of health, psychosocial workload and family life: a cross-sectional study.* BMJ Open, 2012. **2**(4).
17. North, F., et al., *Explaining socioeconomic differences in sickness absence: the Whitehall II Study.* BMJ, 1993. **306**(6874): p. 361-6.
18. Folkhälsomyndigheten, *Förekomst av covid-19 i olika yrkesgrupper inom skolan*, Folkhälsomyndigheten, Editor. 2020: Stockholm.
19. Gohar, B., M. Lariviere, and B. Nowrouzi-Kia, *Sickness absence in healthcare workers during the COVID-19 pandemic.* Occup Med (Lond), 2020. **70**(5): p. 338- 342.
20. Försäkringkassan. *Om läkarintyg Coronaviruset-det här gäller 2020*. 2020 18 May 2021]; Available from: [https://www.forsakringskassan.se/privatpers/coronaviruset- det-har-galler/om-lakarintyg-for-dig-som-ar-arbetsgivare.](https://www.forsakringskassan.se/privatpers/coronaviruset-det-har-galler/om-lakarintyg-for-dig-som-ar-arbetsgivare)
21. AFA-insurance, *Allvarliga arbetsskador och långvarig sjukfrånvaro – 2020*, AFA- insurance., Editor. Juni 2020: Stockholm, Sweden.
22. Allebeck, P. and A. Mastekaasa, *Swedish Council on Technology Assessment in Health Care (SBU). Chapter 5. Risk factors for sick leave - general studies.* Scand J Public Health Suppl, 2004. **63**: p. 49-108.
23. Gohar, B., et al., *Understanding sickness absence in nurses and personal support workers: Insights from frontline staff and key informants in Northeastern Ontario.* Work, 2020. **66**(4): p. 755-766.
24. Lindahl, J.F., et al., *High seroprevalence of SARS-CoV-2 in elderly care employees in Sweden.* Infect Ecol Epidemiol, 2020. **10**(1): p. 1789036.
25. Rydenfalt, C., et al., *Picking low hanging fruit - A scoping review of work environment related interventions in the home care sector.* Home Health Care Serv Q, 2020. **39**(4): p. 223-237.
26. Zhang, J., et al., *The relationship between resilience, anxiety and depression among patients with mild symptoms of COVID-19 in China: A cross-sectional study.* J Clin Nurs, 2020. **29**(21-22): p. 4020-4029.
27. Nishiura, C., et al., *Age-, sex-, and diagnosis-specific incidence rate of medically certified long-term sick leave among private sector employees: The Japan Epidemiology Collaboration on Occupational Health (J-ECOH) study.* J Epidemiol, 2017. **27**(12): p. 590-595.
28. Sakakibara, S., et al., *Predictive factors of the duration of sick leave due to mental disorders.* Int J Ment Health Syst, 2019. **13**: p. 19.
29. Lehnert, T., et al., *Sick leave days and costs associated with overweight and obesity in Germany.* J Occup Environ Med, 2014. **56**(1): p. 20-7.
30. Keramat, S.A., et al., *Gender differences in the longitudinal association between obesity, and disability with workplace absenteeism in the Australian working population.* PLoS One, 2020. **15**(5): p. e0233512.
31. Yang, Q., et al., *Association between vein diameters, reflux characteristics, and clinical severity in patients with chronic venous insufficiency in Northwest China.* J Vasc Surg Venous Lymphat Disord, 2021. **9**(2): p. 401-408 e1.
32. Kompaniyets, L., et al., *Body Mass Index and Risk for COVID-19-Related Hospitalization, Intensive Care Unit Admission, Invasive Mechanical Ventilation, and Death - United States, March-December 2020.* MMWR Morb Mortal Wkly Rep, 2021. **70**(10): p. 355-361.
33. Bhasin, A., et al., *Is BMI Higher in Younger Patients with COVID-19? Association Between BMI and COVID-19 Hospitalization by Age.* Obesity (Silver Spring), 2020. **28**(10): p. 1811-1814.
34. SeyedAlinaghi, S., et al., *Predictors of the prolonged recovery period in COVID-19 patients: a cross-sectional study.* Eur J Med Res, 2021. **26**(1): p. 41.
35. Zheng, X., et al., *Risk factors for the COVID-19 severity and its correlation with viral shedding: A retrospective cohort study.* J Med Virol, 2021. **93**(2): p. 952-961.
36. Westerlind, E., et al., *Patterns and predictors of sick leave after Covid-19 and long Covid in a national Swedish cohort.* BMC Public Health, 2021. **21**(1): p. 1023.
37. Boot, C.R., et al., *Prediction of long-term and frequent sickness absence using company data.* Occup Med (Lond), 2017. **67**(3): p. 176-181.
38. Notenbomer, A., et al., *Predicting long-term sickness absence among employees with frequent sickness absence.* Int Arch Occup Environ Health, 2019. **92**(4): p. 501-511.

Figure/table legends

**Table 1:** Socio-demographic and clinical characteristics of the healthcare and home/service care workers divided into two groups as: sick leave ≤ 3 weeks and sick leave > 3 weeks. P- values <0.05 are of significance. BMI – missing data for 206 subjects.

**Figure 1:** The number of subjects with sick leave for ≤ 3 weeks and > 3 weeks divided into occupational groups and their association with longer sick leave, presented by relative risk (RR) with confidence interval (95% CI). The RR values were adjusted for age, gender, and comorbidity.

Table and figure	[Click here to access/download;Figure;210623_Table figure korr.docx](https://www.editorialmanager.com/pone/download.aspx?id=29369441&amp;guid=e0bb9898-c364-43a1-b0bc-24c10b05caba&amp;scheme=1)

**Table 1:** Socio-demographic and clinical characteristics of the healthcare and home/service care workers divided into two groups as: sick leave ≤3 weeks and sick leave >3 weeks. The P- values <0.05 of significance. BMI – missing data for 206 subjects.

|  | **≤ 3 weeks n=373 (** | **>3 weeks n=60** | **P** |
| --- | --- | --- | --- |
| **Sex (women)** | 283 (84.5) | 52 (15.5) | 0.06 |
| **Age, mean (SD)** | 40.44  (13.30) | 42.34  (12.08) | 0.07 |
| **Occupational group** |  |  |  |
| Physician | 42 (82.3) | 9 (17.7) | 0.40 |
| Nurse/Midwife | 114 (90.5) | 12 (9.5) | 0.12 |
| Assistant nurse | 87 (89.7) | 10 (10.3) | 0.25 |
| Physio- and occupational therapist | 8 (80.0) | 2 (20.0) | 0.63 |
| Psychologist/ curator | 6 (85.7) | 1 (14.3) | 0.99 |
| Biomedical analytics | 6 (85.7) | 1 (14.3) | 0.99 |
| No patient contact | 54 (87.8) | 6 (12.2) | 0.42 |
| Residential care | 53 (74.6) | 18 (25.4) | **<0.01** |
| **Comorbidity** |  |  |  |
| Hypertension | 34 (87.2) | 5 (12.8) | 0.71 |
| Diabetes | 8 (80.0) | 2 (20.0) | 0.63 |
| Hypo-/hyperthyroidism | 29 (90.6) | 3 (0.4) | 0.59 |
| Heart disease | 18 (81.8) | 4 (18.2) | 0.52 |
| Lung disease | 35 (85.3) | 6 (14.7) | 0.87 |
| Cancer/Immunosuppressive treatment | 15 (100) | 0 | 0 |
| Depression/anxiety | 87 (76.9) | 26 (23.1) | **<0.01** |
| **Comorbidity** |  |  |  |
| No disease | 208(88.5) | 27(11.5) | 0.12 |
| One disease | 111(83.5) | 22(16.5) | 0.27 |
| Two or more diseases | 54(83.1) | 11(16.9) | 0.42 |
| **BMI** |  |  |  |
| Underweight <18.5 | 3 (60.0) | 2 (40.0) | 0.17 |
| Normal weight 18.5-25 | 102 (87.2) | 15 (12.8) | 0.26 |
| Overweight >25-30 | 62 (87.3) | 9 (12.7) | 0.44 |
| Obesity >30 | 25 (73.5) | 9 (26.5) | 0.05 |
| **Symptom at onset** |  |  |  |
| Fever | 174 (84.5) | 32 (15.5) | 0.33 |
| Dyspnea | 65 (73.8) | 23 (26.2) | **<0.01** |
| Muscle/joint pain | 173 (83.2) | 35 (16.8) | 0.85 |
| Impaired taste and smell | 93 (94.9) | 5 (5.1) | **<0.01** |
| Sore throat | 110 (83.3) | 22 (16.7) | 0.29 |
| Headache | 157 (84.8) | 28 (15.2) | 0.50 |
| Nasal symptoms | 253 (85.2) | 44 (14.8) | 0.39 |
| GI symptoms | 23 (82.1) | 5 (17.9) | 0.56 |
| Fatigue | 90 (91.8) | 8 (8.2) | 0.06 |
| Pressure over chest | 24 (80.0) | 6 (20.0) | 0.31 |
| **The length of sick leave prior to the pandemic (January 2019-February 2020)** |  |  |  |
| <3 week | 39 (70.9) | 16 (29.1) | 0.49 |
| 3–12 weeks | 27 (73.0) | 10 (27.0) | 0.24 |
| >12 weeks | 9 (75.0) | 3 (25.0) | 0.58 |

**Figure 1:** The number of subjects with sick leave for ≤3 weeks and >3 weeks divided into occupational groups and their association with longer sick leave presented by relative risk (RR) with confidence interval (95% CI). The RR values were adjusted for age, gender, and comorbidity.

Supplementary material

[Click here to access/download](https://www.editorialmanager.com/pone/download.aspx?id=29369443&amp;guid=c4f7e07e-21f0-4180-8e1c-1c3140711968&amp;scheme=1)

[**Supporting Information** 210623_Suppelementary materials korr.docx](https://www.editorialmanager.com/pone/download.aspx?id=29369443&amp;guid=c4f7e07e-21f0-4180-8e1c-1c3140711968&amp;scheme=1)
